# Aging‐associated atrial fibrillation: A comprehensive review focusing on the potential mechanisms

**DOI:** 10.1111/acel.14309

**Published:** 2024-08-12

**Authors:** Meng‐Fei Wang, Can Hou, Fang Jia, Cheng‐Hao Zhong, Cong Xue, Jian‐Jun Li

**Affiliations:** ^1^ The Third Affiliated Hospital of Soochow University The First People's Hospital of Changzhou Changzhou China; ^2^ State Key Laboratory of Cardiovascular Diseases, Fu Wai Hospital, National Center for Cardiovascular Diseases Chinese Academy of Medical Sciences and Peking Union Medical College Beijing China

**Keywords:** aging, atrial fibrillation, mechanisms

## Abstract

Atrial fibrillation (AF) has been receiving a lot of attention from scientists and clinicians because it is an extremely common clinical condition. Due to its special hemodynamic changes, AF has a high rate of disability and mortality. So far, although AF has some therapeutic means, it is still an incurable disease because of its complex risk factors and pathophysiologic mechanisms, which is a difficult problem for global public health. Age is an important independent risk factor for AF, and the incidence of AF increases with age. To date, there is no comprehensive review on aging‐associated AF. In this review, we systematically discuss the pathophysiologic evidence for aging‐associated AF, and in particular explore the pathophysiologic mechanisms of mitochondrial dysfunction, telomere attrition, cellular senescence, disabled macroautophagy, and gut dysbiosis involved in recent studies with aging‐associated AF. We hope that by exploring the various dimensions of aging‐associated AF, we can better understand the specific relationship between age and AF, which may be crucial for innovative treatments of aging‐associated AF.

## Graphical abstract

Our review highlights the potential mechanisms of aging‐associated atrial fibrillation, and in particular describes the potential relationship between new aging features and aging‐associated atrial fibrillation, including mitochondrial dysfunction, telomere attrition, cellular senescence, macroautophagy dysfunction, and gut dysbiosis, to deepen our understanding of the mechanisms of aging‐associated atrial fibrillation and to contribute to the individualized therapeutic strategies for elderly patients with atrial fibrillation.

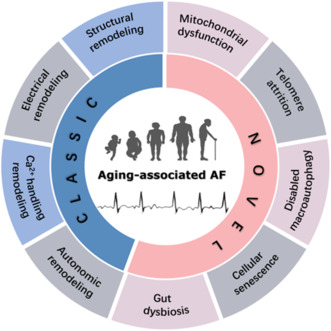

**Abbreviations**
AF: atrial fibrillation
ANS: autonomic nervous system
APD: action potential duration
ARGP: anterior right ganglionated plexi
BAs: bile acids
CaMKII: calmodulin‐dependent protein kinase II
CDCA: chenodeoxycholic acid
EADs: early afterdepolarizations
ERP: effective refractory period
GP: ganglionated plexi
GPR43: G‐protein‐coupled receptor 43
GPR43: G‐protein‐coupled receptor 43
H_2_O_2_
: hydrogen oxide
IL‐1R: interleukin‐1 receptor
IL‐1β: interleukin‐1β
IL‐6: interleukin‐6
LPS: lipopolysaccharide
MMPs: matrix metalloproteinases
NF‐κB: nuclear factor kappa B
NGF: nerve growth factor
NLRP3: NACHT, LRR, and PYD domains‐containing protein 3
PAGln: phenyl acetyl glutamine
ROS: reactive oxygen species
RyR2: ryanodine receptor isoform 2
SASP: senescence‐associated secretory phenotype
SCFAs: short‐chain fatty acids
SERCA2a: SR Ca^2+^‐ATPase type 2a
SIRT1: NAD^+^‐dependent deacetyase sirtuin 1
SR: sarcoplasmic reticulum
SR: sarcoplasmic reticulum
TAMO: trimethylamine N‐oxide
TGF‐β: transforming growth factor‐β
TLR4: toll‐like receptor 4

## 1 INTRODUCTION

Atrial fibrillation (AF) is one of the most common forms of arrhythmia in clinical practice, and patients with AF can be at risk of cognitive decline, ischemic stroke, heart failure, myocardial infarction, and even death due to atrial and ventricular contraction asynchrony, hemodynamic alterations, and thromboembolism (Benjamin et al., 2021). An estimated 46.3 million people worldwide suffered from AF in 2016 (Virani et al., 2020); thus, AF is becoming one of the major health burdens worldwide. In addition, during the 21‐year period from 1993 to 2013, hospitalizations of patients with AF increased by 295% and hospitalization costs incurred by patients with AF increased by 479%, both of which far exceed those of patients with heart attacks and heart failure (Gallagher et al., 2019). These data further emphasize the importance of studying the pathophysiology of AF. AF is commonly viewed as a progressive disease that typically transitions from a paroxysmal to a persistent form and eventually to a long‐term persistent (chronic or permanent) form. Nevertheless, not all individuals progress through all stages, and the duration spent in each stage can differ significantly (Heijman et al., 2014). Current antiarrhythmic treatments include drugs, radiofrequency ablation, and surgery (Heijman et al., 2015), and although these approaches have largely improved clinical outcomes, their efficacy remains suboptimal. After decades of research, AF is recognized as a multifactorial disease characterized by sex, age, body mass index, and comorbidities such as hypertension, metabolic syndrome, heart failure, myocardial infarction, and sleep apnea, all of which are well‐defined risk factors for the development and persistence of AF (Kornej et al., 2020). AF is also a disease with multiple complex mechanisms, and its underlying mechanisms have been reported by an increasing number of studies. These mechanisms include structural remodeling, electrical remodeling, abnormal Ca^2+^ handling, autonomic dysfunction, and genetic factors (Nattel & Harada, 2014). Because AF is so complex, it remains a disease that humans have failed to overcome.

**FIGURE 1:**
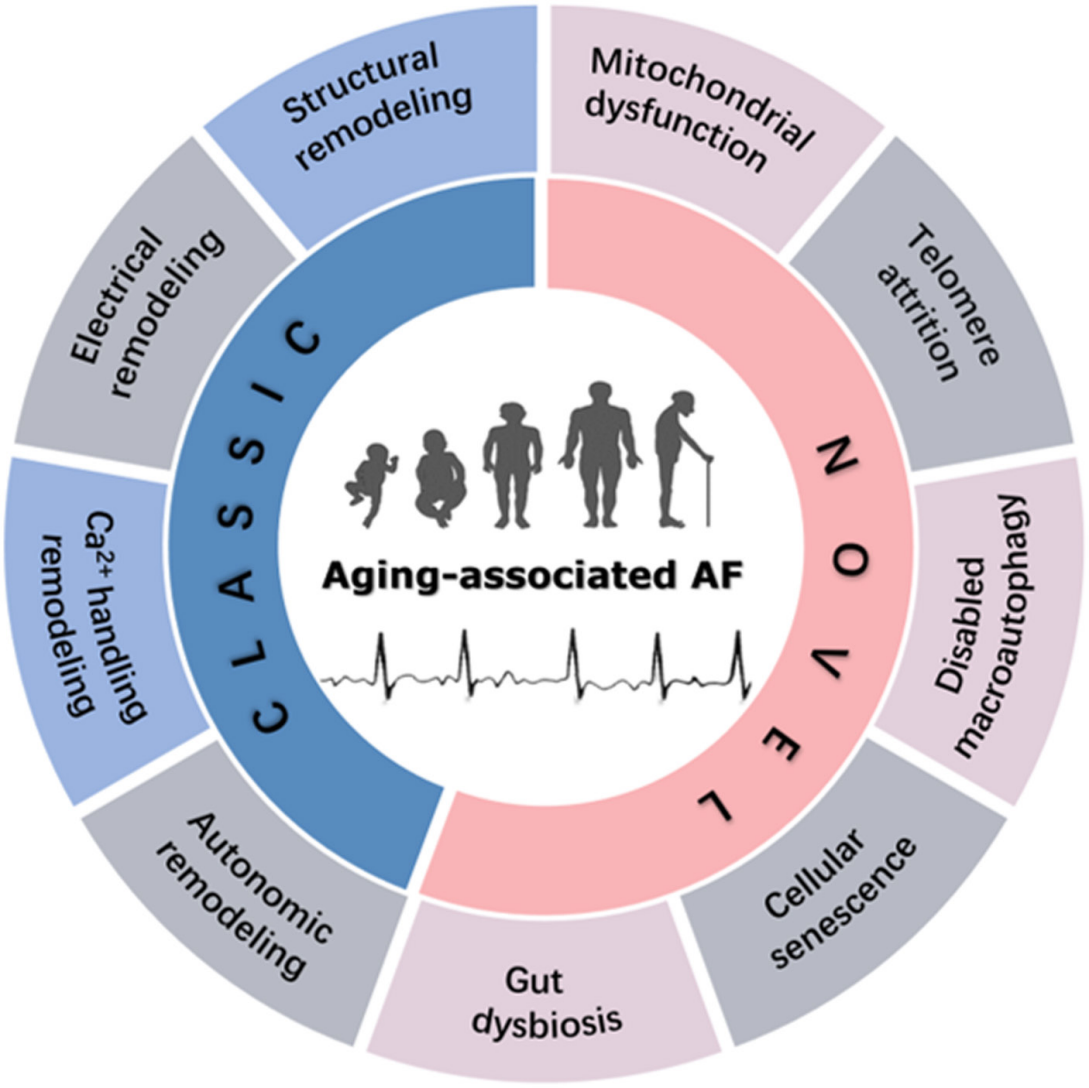
Potential mechanisms of aging‐associated AF. Mechanisms such as Electrical remodeling, structural remodeling, Ca^2+^ handling remodeling, mitochondrial dysfunction, telomere attrition, disabled macroautophagy, cellular senescence, and gut dysbiosis are categorized into classic and novel categories. AF, atrial fibrillation.

Accumulating evidence indicates that aging is an important independent risk factor for AF and that the prevalence of AF increases with age (Kornej et al., 2020), even in the absence of comorbidities. The prevalence of AF has been reported to increase from 0.1% to 9.0% in adults under 55 years of age to those aged 80 years or older (Go et al., 2001). With the increasing prevalence of population aging, ongoing efforts are investigating the pathophysiological mechanisms of aging in human heart disease (Dai et al., 2012). Indeed, the link between AF and aging is currently under intensive investigation (Gao et al., 2022; Ravassa et al., 2019; Roberts et al., 2021). Here, we review the classical pathophysiological evidence for aging‐associated AF. We then explore novel pathophysiological mechanisms of aging‐associated AF that have been addressed in recent studies (Figure 1). We conclude with the hope that our review may provide new insights into the prevention and treatment of patients with aging‐associated AF.

## 2 CLASSIC POTENTIAL MECHANISMS OF AGING‐ASSOCIATED AF

### 2.1 Structural remodeling

Aging induces cardiac remodeling even without underlying pathology (Bradshaw et al., 2010). With advancing age, the heart undergoes several structural alterations at the cellular level. Specifically, the endocardial cells thicken and exhibit areas of opacity, most notably in the left atrium (LA). The prevalent structural alteration of the epicardium involves the accumulation of adipocyte cells along the right ventricle and left ventricle, accompanied by regions of fibrosis. Age‐related changes also include a reduction in the total cardiomyocyte count due to autophagy and apoptosis, with the surviving cardiomyocytes undergoing compensatory hypertrophy. Furthermore, the aging heart manifests fibroblast proliferation, collagen dysregulation, and interstitial fibrosis in both the atria and ventricles. The functional integrity of the conduction system is compromised by collagen deposition, adipose tissue infiltration, amyloidosis, and fibrosis, culminating in an arrhythmogenic state (Ribeiro et al., 2023). Aging is associated with significant atrial structural changes, especially atrial fibrosis, which plays a vital role in the structural remodeling of the atria (Burstein & Nattel, 2008). The increased size of the LA with aging is the primary biological indicator of AF and a determinant of the presence of persistent AF (Pan et al., 2008; Singh et al., 2022). In addition, a low atrial voltage is considered a surrogate marker of cardiac fibrosis, and low‐voltage areas are a consequence of structural remodeling of the LA, contributing to the progression and maintenance of AF (Nattel et al., 2008; Yamaguchi et al., 2022). Recently, a large cohort study revealed that low‐voltage areas of the heart were strongly associated with higher age (Huo et al., 2017). Furthermore, the relationship between atrial fibrosis and age has also been shown in human pathological tissues from patients who underwent cardiac surgery and who had a history of comorbid AF (Goette, 2002). In a canine model, the amount of fibrous tissue in aged atria tripled, and this fibrosis was universally distributed in both atria (Anyukhovsky, 2002). However, in a rat study, aging‐associated fibrosis differed between the right and left atria (Swartz et al., 2009).

Commonly, atrial fibrosis underlies atrial conduction abnormalities, and this fibrosis underlies the persistence of AF (Kottkamp, 2013). Aging‐associated fibrosis exhibits an inhomogeneous and anisotropic distribution in the atria, and this fibrosis first affects lateral electrical conduction. In electrophysiology, this change results in a pronounced zigzag propagation process, which contributes to slowing the speed of propagation in the horizontal direction, thereby promoting the initiation of reentry (Spach & Dolber, 1986). In a dog model, atrial fibrosis led to localized regional conduction slowing and increased conduction heterogeneity, providing an electrical basis for the development of AF (Li et al., 1999). Human histopathological analyses and recent magnetic resonance imaging studies have shown that aging‐associated atrial fibrosis affects the posterior wall of the LA first, which is consistent with the understanding that the sustaining foci of AF are located in this region (Benito et al., 2018). Fibrosis and enlargement of the atria provide the structural basis for the occurrence of AF. Some scholars have suggested that chronic atrial enlargement may facilitate the structural remodeling and taming of AF (Schotten et al., 2003). In addition to its fibrogenic role, the mechanical stretching of fibroblasts can also regulate the electrical activity of muscle cells through a process known as mechanoelectric feedback (Kamkin et al., 2005). Therefore, reducing the mechanical pulling of the atria and pulmonary veins (PV) to lower the sensitivity of patients to AF has emerged as a novel approach in antiarrhythmic therapy (Gottlieb et al., 2023).

In addition to fibrosis, a variety of nonfibrotic structural changes may be involved in the development of aging‐associated AF. Epicardial adipose tissue (EAT) is a biologically active organ that is adjacent to the myocardium, has no fascial border, and causes paracrine effects by releasing adipokines. Aging and obesity lead to EAT hypertroph (Hatem & Sanders, 2014; Willar et al., 2023). Increased EAT is independently associated with AF and recurrence after AF ablation (Kawasaki et al., 2020; Le Jemtel et al., 2019). Several studies have shown that EAT induces atrial myocardial fibrosis through the adipose secretion of fibrotic factors (Venteclef et al., 2014). The EAT encompasses not only adjacent atrial cardiomyocytes but also a robust vascular stromal component and envelops the ganglionic plexus associated with the cardiac autonomic nervous system (cANS). The pivotal role of the cANS in the development of AF is well established, suggesting a reciprocal interaction between the EAT and the cANS (Willar et al., 2023). This complex interaction may be critical for the development of AF. Age‐related amyloidosis is also another aspect of aging‐related atrial structural remodeling. Amyloid deposition affects all cardiac chambers, especially the atria, and atrial amyloidosis has also been implicated as a potential substrate for AF (Röcken et al., 2002). Atrial cardiomyocyte hypertrophy alters cellular Ca^2+^ transients, which may promote AF (Zhang et al., 2015).

### 2.2 Electrical remodeling

Some studies have examined electrical changes in the atria associated with aging, but results have been inconsistent. In several animal models, studies utilizing conventional microelectrode recordings and diaphragm clamp methods have repeatedly shown a prolongation of the action potential duration (APD) and increased dispersion of the atrial effective refractory period (ERP), both of which are favorable for arrhythmogenesis (Anyukhovsky et al., 2005; Huang et al., 2006; Su et al., 1995; Toda, 1980). In a rat study, the APD and ERP were found to be prolonged in the senescent right atrium but shortened in the LA. However, as the frequency of stimulation increased, the APD decreased accordingly in both the left and right atrial myocardium, and the shortening of the APD was significantly greater in the right atrial myocardium than in the left atrial myocardium in the aged group (Huang et al., 2006). Some human electrophysiology studies have shown that the atrial ERP increases with age (Brembilla‐Perrot et al., 2004; Sakabe et al., 2003). However, other studies suggest that age may not affect atrial refractoriness, but thess studies are limited by the use of single‐site recordings, the analysis of individuals taking antiarrhythmic drugs, and the lack of participants over the age of 70 in the definition of the elderly population (Brorson & Olsson, 2009; Taneja et al., 2001).

These changes in action potentials (APs) are spatially heterogeneous in the aged atrium (Anyukhovsky et al., 2005), and this spatial variation in the duration of the period may lead to localized regions of transient conduction block, which depend on the relative length of the should period (Allessie et al., 1976). Recent research has shown that regions with prolonged refractory periods are consistent with areas of conduction block and intramyocardial reentry (Allessie et al., 1976). Therefore, the prolonged APD associated with aging and increased spatial heterogeneity may make the atrium more prone to reentrant arrhythmias. Additionally, the heterogeneity of atrial ERP plays a crucial role in the initiation and duration of AF (Fareh et al., 1998). In humans, increased dispersion of atrial refractoriness is associated with AF (Diker et al., 1998).

Notably, recent ionic mechanisms regarding regional heterogeneity of average adult and aged atria may explain the increased dispersion of various AP parameters in aged atria. In an animal study, regional differences in AP morphology and duration in the right atrium of dogs were found to be caused by variations in I_to_ (transient outward K^+^ current channel), I_Kr_ (ultrafast delayed rectifier K^+^ current channel), I_CaL_ (Ca^2+^ channel), and density (Feng et al., 1998). Another study revealed changes in the inward and outward currents in the right atrial comb muscle in aged dogs (Dun, 2003). They discovered that I_CaL_ was downregulated, and I_to_, I_kr_, and I_sus_ (sustained K^+^ channels current) were upregulated in aging cells. Interestingly, acetylcholine‐induced potassium currents (I_KACh_) mediate parasympathetic responses and are more strongly expressed in aged atria (Su et al., 1995).

These data suggest that changes in ionic currents associated with aging may be spatially inhomogeneous, leading to changes in various parameters of the action potential and increased dispersion. These electrophysiological bases favor the development of AF in aged individuals.

Atrial gap junctions are composed of connexin43 (Cx43) and connexin40 (Cx40), which form specialized membrane channel structures that influence electrical and chemical signal propagation between adjacent myocytes (Dhein & Salameh, 2021). Gap junction Cx43 protein levels are significantly reduced in human and rabbit atria with the age at diagnosis. At the same time, an in vitro cellular model revealed that c‐Jun N‐terminal kinase (JNK) activation reduces gap junction Cx43 levels in cells and impairs intercellular coupling, thereby promoting the development of AF (Yan, Thomson, et al., 2018). In a canine optical mapping study, Cx43 expression decreased with diagnostic age, and its distribution gradually shifted from a uniform distribution over the entire cell surface to the lateral margins of the cell, thus severely affecting lateral conductance (Koura et al., 2002). This age‐related change in structural anisotropy increases the susceptibility of older patients to multiple folds and increases the incidence of AF. Recent studies have also shown that in mouse models, very low‐density lipoproteins induce the downregulation of Cx40 and Cx43 at the transcriptional, translational, and tissue levels, impairing the stability of gap junctions and delaying atrial conduction, thus promoting the development of AF (H.‐C. Lee et al., 2017). Aging decreases the expression of hyperpolarization‐activated cyclic nucleotide‐gated channel 2/4 (HCN2/4) in the sinus node but increases their expression in the atria and PVs, leading to sinus node dysfunction (SND). The reason may be that HCN downregulation favors bradycardia by acting on the sinus node, and the association of SND with AF is well recognized. In contrast, high HCN expression in the atria and PVs produces relatively large If, which increases ectopic electrical activity and promotes atrial arrhythmias (Duan & Du, 2023; Marciszek et al., 2021).

### 2.3 Calcium‐handling remodeling

Previously, aging has been shown to alter the structural, electrical, and mechanical activities of the heart, leading to systolic and diastolic dysfunction, slowing conduction velocity, and altering conduction anisotropy, which provides the basis for proarrhythmic and increases the risk of malignant arrhythmias (Cooper et al., 2012). At the cellular level, aberrant Ca^2+^ handling is thought to be an important contributor to the electrical dysfunction associated with aging (Hamilton & Terentyev, 2019; Xiao et al., 1994). (Cooper et al., 2013) went a step further by isolating ventricular myocytes from the hearts of senescent rabbits and demonstrated that thiol oxidation of ryanodine receptors (RyRs) due to the aging‐associated increase in the rate of reactive oxygen species (ROS) production by mitochondria resulted in the hyperactivation of these channels, which leads to changes in Ca^2+^ homeostasis and an increase in arrhythmias. These authors explained the abnormal Ca^2+^ handling associated with aging at the molecular level. Increased diastolic Ca^2+^ release from the sarcoplasmic reticulum (SR) through ryanodine receptor isoform 2 (RyR2) leakage was found in atrial samples from elderly patients with AF (Hove‐Madsen et al., 2004; Yan, Zhao, et al., 2018), which was accompanied by hyperphosphorylation of phospholamban (El‐Armouche et al., 2006). In a rat model, more significant fibrosis in the atria of aged rats amplifies abnormal Ca^2+^ processing, which promotes early afterdepolarization (EAD)‐mediated triggered activity and AF (Ono et al., 2007).

Abnormal intracellular Ca^2+^ handling may be essential in initiating and maintaining episodes (Nattel & Dobrev, 2012). Experimental studies have found that age‐related abnormalities in Ca^2+^ handling are associated with EAD (Ono et al., 2007) and delayed afterdepolarization (DAD) (Sood et al., 2008). EAD usually occurs in the context of prolonged APD, such as the absence of repolarizing K^+^ currents (Zellerhoff et al., 2009) or an excess of late, unactivated Na^+^ currents (sustained/late I_Na_) (Lemoine et al., 2011). During standard AP, L‐type Ca^2+^ channels limit Ca^2+^ influx through voltage‐ and Ca^2+^‐dependent inactivation. Prolonged APD allows L‐type Ca^2+^ channels to recover from inactivation, leading to inward currents that result in EAD. Spontaneous AF, which occurs via Ca^2+^‐dependent EAD at the LA/PV junction, has been identified in a large animal model of AF (Numata et al., 2011). In another model of aged rats, glycolysis inhibitors interacted with the fibrous matrix of aged atria, amplifying abnormal Ca^2+^ handling and thus promoting EAD‐mediated triggered activity and AF (Ono et al., 2007).

DAD is mainly caused by aberrant SR Ca^2+^ leakage and diastolic SR Ca^2+^ release events (SCaEs) and is facilitated by increased SR Ca^2+^ loading and RyR2 dysfunction (increased phosphorylation and opening rates) (Heijman et al., 2014). In addition to manifesting as RyRs dysfunction, this process can also manifest as improved SR Ca^2+^‐ATPase type 2a(SERCA2a)function, increased Ca^2+^ sparks and Ca^2+^ waves, and enhanced calmodulin‐dependent protein kinase II (CaMKII) function (followed by hyperphosphorylation of RyRs) (Kistamás et al., 2020). The diastolic release of Ca^2+^ from the SR activates the Na^+^/Ca^2+^ exchanger (NCX), which generates a transient inward current that leads to membrane depolarization. If DAD reaches a threshold, an ectopic AP is triggered (Heijman et al., 2014). Repeated focal ectopic activity can sustain AF even without AF‐sustaining substrates (Alhede et al., 2016). In addition, focal ectopic discharges can initiate the reentry circuit of AF. The reentry mechanism is the primary mechanism that sustains AF, and it can occur around anatomical barriers or functional blocks (Heijman et al., 2015).

A new and interesting theory of Ca^2+^ signaling silencing has proposed that it serves as an “antiarrhythmic” mechanism in AF patients themselves (Greiser et al., 2014). Underlying cardiovascular disease (e.g., heart failure, ischemic heart disease) likely produces Ca^2+^‐based arrhythmogenic substrates, and Ca2 + −based arrhythmogenic mechanisms contribute to the development of AF in these patients; however, some degree of Ca^2+^ silencing occurs after the onset of AF, at which point Ca^2+^‐based arrhythmogenesis may not further contribute to the maintenance of AF. Similarly, in patients without concomitant cardiac disease (“isolated AF”), Ca2 + −based arrhythmias may not play a role in the development and maintenance of AF (Greiser, 2017). Consistent with this concept, sustained high atrial rate pacing also does not necessarily lead to Ca^2+^ destabilization. Specifically, the amount of intracellular Ca^2+^ sparks and arrhythmogenic Ca^2+^ waves, as well as Ca^2+^ spark‐mediated SR Ca^2+^ leakage, did not change after rapid atrial pacing. The key feature of Ca^2+^ signaling silencing is the inability of Ca^2+^ signals to propagate to the center of the cell. The authors suggested that the “phenotype” of Ca^2+^ signaling in patients with AF is the net result of stabilization (silencing of Ca^2+^ signaling) and destabilization (arrhythmogenic Ca^2+^ destabilization) (Greiser et al., 2014). Indeed, the most recent review summarized the critical role of intracellular Na^+^ homeostasis in Ca^2+^ processing in AF, suggesting that Na^+^ blockers could be a new target for the treatment of AF (Kaplan et al., 2021).

Therefore, numerous studies indicate that changes in Ca^2+^ handling associated with aging contribute to the onset and maintenance of AF by remodeling cellular electrophysiology and promoting ectopic activity.

### 2.4 Autonomic remodeling

The cANS comprises both extrinsic and intrinsic elements (Armour, 2010). Extrinsic innervation, which is divided into sympathetic and parasympathetic branches, generally governs cardiac activity through coordinated interplay (Hasan, 2013). Within the sympathetic nervous system, acetylcholine serves as the primary neurotransmitter in preganglionic fibers, while norepinephrine is predominant in postganglionic fibers. Norepinephrine and epinephrine stimulate various adrenergic receptor subtypes—α1, α2, and β—with β receptors specifically present in cardiac myocytes (CM) (Triposkiadis et al., 2009). Activation of cardiac β receptors enhances chronotropy, induces tachycardia, increases inotropy, and promotes relaxation. Conversely, parasympathetic fibers synapse within the cardiac ganglia situated in the heart, extending short postsynaptic fibers to the myocardium. These ganglia are embedded in the epicardial fat of the atrial and ventricular walls and within the major cardiovascular vessel plexuses (Hasan, 2013). Parasympathetic neurons primarily utilize acetylcholine, which acts on muscarinic receptors (M1‐5), including M2 and M3 receptors found in the heart (Wang et al., 2006). Activation of the cardiac parasympathetic system typically leads to decreased chronotropy, reduced heart rate variability, and diminished positive inotropy.

The aging myocardium exhibits decreased β‐adrenergic receptor responses, β‐adrenoreceptor desensitization, and decreased β‐adrenergic receptor density (Ferrara et al., 2014). Thus, any increase in cardiac function after β‐adrenergic stimulation diminishes with age. In contrast to the sympathetic nervous system, little is known about the effects of aging on the parasympathetic nervous system. However, the density and function of M2 receptors are thought to decline with age, which may lead to a decrease in cardiac parasympathetic activity, which is partly attributed to a decrease in stress reflex activity with age (Chadda et al., 2018). Some findings further reveal an age‐related decline in parasympathetic function, with a decrease in the vagal component of heart rate variability with age and a diminished heart rate response to muscarinic acetylcholine receptor blockade (Poller et al., 1997).

Both sympathetic and parasympathetic stimulation have been implicated in the pathogenesis of AF (Tan et al., 2008). The underlying mechanisms are thought to be because sympathetic activation can alter L‐type Ca^2+^ currents, increase RyR2 opening rates, and increase SR Ca^2+^ leakage through CaMK II and protein kinase phosphorylation, whereas parasympathetic activation decreases the atrial ERP. In summary, the mismatch between intracellular Ca^2+^ transients and the ERP results in an increase in DAD and ectopic discharges (Nattel & Harada, 2014; Shen & Zipes, 2014). Selective ablation of intrinsic cardiac ganglia around PVs decreases discharge and prolongs the atrial ERP, thereby decreasing the likelihood of AF (Wake & Brack, 2016).

## 3 NOVEL POTENTIAL MECHANISMS OF AGING‐ASSOCIATED AF

### 3.1 Mitochondrial dysfunction

Mitochondria are a source of cellular energy and a potential trigger for inflammation and cell death (López‐Otín & Kroemer, 2021). With aging, mitochondrial function declines, primarily due to a combination of factors such as the accumulation of mutations in mitochondrial DNA (mtDNA), inadequate protein stabilization affecting respiratory chain complex stability, diminished organelle renewal, and changes in mitochondrial dynamics. These conditions impair the mitochondrial supply of cellular energy, increase ROS production, and may trigger the unintended permeabilization of the mitochondrial membrane, leading to inflammation and cell death (Amorim et al., 2022). Mitochondrial function is a significant marker for maintaining organismal health, and a deterioration of mitochondrial function can contribute to the aging phenotype. Lifespan extension interventions can promote mitochondrial function; indeed, lifespan can be prolonged by improving mitochondrial function (López‐Otín, Blasco, et al., 2023).

Various studies have investigated the role of mitochondrial dysfunction in AF (Gambardella et al., 2017). In atrial cardiomyocytes, oxidative stress and mtDNA damage are increased in patients with AF (Lin et al., 2003), which leads to mitochondrial dysfunction and the onset and progression of AF (Bukowska et al., 2008; Xie et al., 2015). Furthermore, in experimental and clinical AF, decreased ATP levels, deficits in the mitochondrial membrane potential, and a disrupted mitochondrial network have been observed to lead to contractile dysfunction and the progression of AF (Wiersma et al., 2019). These findings suggest that mitochondrial dysfunction is central to the pathophysiological mechanisms driving AF, especially in aged patients.

The mitochondrion is an essential organelle in the metabolism that maintains cardiac function (Palaniyandi et al., 2010). It synthesizes the ATP used to support the electrical and mechanical activities of the heart (Murphy et al., 2016). Mitochondria produce ATP and another byproduct of biological energy activity—ROS (Chen et al., 2003), including hydrogen peroxide (H_2_O_2_), hydroxyl radicals, and peroxynitrite, etc (Sovari & Dudley, 2012). Usually, this byproduct is neutralized or scavenged by antioxidants. If the production rate and the ability to scavenge ROS are essentially constant and balanced, tissues and cells are healthy (Dröge, 2002). However, excessive oxidative stress leads to impaired antioxidant defenses. This impairment increases ROS production, causes cellular damage, and contributes to the development of aging‐associated heart disease (Taverne et al., 2013).

Emerging studies have also suggested that excess ROS directly affects ion channels and action potential propagation (Sovari & Dudley, 2012).H_2_O_2_ induces APD prolongation and the formation of EAD by augmenting late Na^+^ currents (Song et al., 2006). ROS can induce the downregulation of total Na^+^ currents, which promotes the formation of foldback circuits. This phenomenon can be reversed by mitochondrial antioxidants (Liu et al., 2010). Notably, ROS can directly upregulate L‐type Ca^2+^ currents and promote EAD by altering intracellular calcium homeostasis (Sovari & Dudley, 2012). Excess ROS also increases I_to_ currents (Sridhar et al., 2009). Therefore, not surprisingly, ROS contribute to the progression of AF by altering APs through their effects on ion channels, causing unstable electrical activity. Another mechanism by which ROS exert their proarrhythmic effects is by oxidizing CaMKII (Yoo et al., 2018) and RyR2 (Xie et al., 2015), leading to abnormal Ca^2+^ release, which contributes to the onset and progression of AF. The production of ROS in the myocardium is attributed to several enzymatic sources. Among these enzymes, NADPH oxidase (NOX) has been shown to play a crucial role in the progression of AF. In studies of animal models, superoxide and H_2_O_2_ produced by activated NOX2 and NOX4 isoforms lead to myocyte apoptosis, fibrosis, inflammation, and ion channel alterations, which further contributed to the persistence of AF (Youn et al., 2013). However, this electrical remodeling of AF caused by NOX oxidative damage can be successfully prevented through genetic methods (Yoo et al., 2020). Monoamine oxidases (MAOs) are a class of enzymes critical for the regulation of catecholamines and other biogenic amines, as well as major producers of mitochondrial ROS (Santin et al., 2021). MAO has been shown to correlate with an increased risk of AF in the postoperative period and is an important determinant of myocardial redox homeostasis in the human atrium (Anderson et al., 2014). Notably, in addition to electrical remodeling stimulated by the above mechanisms, ROS also contributes to atrial structural remodeling. Hydroxyl radicals can alter the structure and function of myofibrillar proteins, promoting myocardial injury and further contributing to the formation of a conducive environment for arrhythmias (Babušíková et al., 2004).

Recent studies have confirmed that mitochondria and the SR are well connected through a network of microtubules (Brette & Orchard, 2003; Franzini‐Armstrong et al., 2005). In cardiomyocytes, microtubule stability affects mitochondrial function and Ca^2+^ homeostasis, which are essential for ensuring myocardial contractility and function (Hammond et al., 2008). The microtubule network consists mainly of α‐ and β‐microtubule proteins, and they are regulated primarily by posttranslational modifications of microtubule protein subunit repair (Janke & Magiera, 2020). Acetylated α‐microtubulin governs the stability and function of the microtubule network. Microtubulin interacts with voltage‐dependent anion channels (VDACs) on the outer mitochondrial membrane (OMM) (Rostovtseva et al., 2008), which play a key role in controlling efficient signaling between the two cell types, mitochondrial function, and cardiac contraction (Murley & Nunnari, 2016). The activity of VDACs and the mitochondrial membrane potential are regulated by respiratory substrates, including NADH (Zizi et al., 1994), and ATP (Rostovtseva & Colombini, 1996). In summary, the function of mitochondria is closely related to the microtubule network and respiratory substrates.

Loss of connectivity between organelles leads to Ca^2+^ overload in mitochondria and results in mitochondrial dysfunction and myocardial contractile dysfunction (Santulli et al., 2015). Additionally, evidence to suggest that microtubule disruption may be critical for the development of AF (Hu et al., 2019). Intercellular metabolite transport and myocardial contractile function are mediated by acetylated α‐microtubule proteins (Zhang et al., 2019). In cardiomyocytes, histone deacetylase 6 (HDAC6) overexpression leads to the deacetylation and degradation of α‐microtubulin (Hubbert et al., 2002). Increased deacetylation and increased degradation of α‐microtubulin have been observed in the atria of patients with persistent AF. Furthermore, HDAC6‐induced deacetylation and degradation of the microtubule network may underlie mitochondrial dysfunction, as observed in experimental and clinical AF (Zhang et al., 2019). In conclusion, the disruption of the microtubule network observed in subjects with AF may be a key regulator leading to mitochondrial dysfunction and the progression of AF, especially in elderly patients.

Mitochondria and the SR are physically connected through the microtubule network and Mitofusin 2 (Mfn2) protein‐mediated fusion, thus allowing Ca^2+^ cross‐talk between the two organelles via VDACs (Naon et al., 2016). VDACs contain Ca^2+^‐binding sites and are therefore highly permeable to Ca^2+^ and regulate mitochondrial intermembrane Ca^2+^ levels (Konstantinidis et al., 2012). Mitochondrial input of SR‐derived Ca^2+^ depends on a suitable physical distance between the two organelles (Csordás et al., 2010). In contrast, lack of Mfn2 interferes with Ca^2+^ conductance between mitochondria and the SR, leading to myocardial oxidative stress (Chen et al., 2012). Because of the importance of the connection between mitochondria and SR for Ca^2+^ transient processing, the finding that the disruption of the microtubule network or Mfn2 impairs Ca^2+^ processing, myocardial contraction, and mitochondrial function is not surprising (Denham et al., 2018; Dibb et al., 2009). Thus, protection of the physical connection between the two organelles is essential to ensure atrial cardiomyocyte function.

Briefly, mitochondrial dysfunction plays a role in the development of AF through multiple pathways, mainly through the effects of ROS on electrical remodeling, Ca^2+^ handling, and structural remodeling, as well as through the impact of the connection between mitochondria and the SR on Ca^2+^ handling, which is closely linked with aging‐associated AF.

### 3.2 Telomere attrition

Telomeres are repetitive nucleoprotein structures located at the ends of chromosomes. Damage to telomeres leads to aging and aging‐related diseases (Blackburn et al., 2015). Replicative DNA polymerases cannot complete copies of the telomeric regions of eukaryotic DNA. Consequently, after several rounds of cell division, telomeres shorten significantly and induce genomic instability, leading to apoptosis or cellular senescence. The reverse transcriptase activity of telomerase prevents these harmful effects, which lengthens telomeres to maintain their adequate length (Blasco, 2005; Chakravarti et al., 2021). However, most mammalian somatic cells do not express telomerase, resulting in the cumulative impairment of telomere sequences (López‐Otín, Pietrocola, et al., 2023). Telomere shortening has been observed in many normally aging species, and the rate of telomere shortening has even been found to be a potential predictor of longevity. Whittemore et al. obtained telomere length and telomere shortening rates in birds and a number of mammals (including mice, goats, reindeer, and bottlenose dolphins) using the high‐throughput quantitative fluorescence in situ hybridization (HTQ‐FISH) technique. They found that species longevity was not correlated with the initial telomere length, while the telomere shortening rate was a strong predictor of species longevity. The greater the shortening of telomeres each year, the shorter the lifespan of the species (Whittemore et al., 2019). Pharmacological activation of telomerase extends the lifespan of normally aging mice (Bernardes de Jesus et al., 2012), and even mice with ultralong telomeres have an extended lifespan and improved metabolism (Muñoz‐Lorente et al., 2019).

Telomere dysfunction is associated with many diseases involving various organ systems throughout the body, including the lungs, blood, kidneys, skeleton, and heart (Rossiello et al., 2022). Telomere damage and shortening have been reported as markers and potential driving factors of heart disease and as indicators of treatment outcomes (Boniewska‐Bernacka et al., 2020). Similarly, many studies have explored the relationship between aging‐associated AF and telomeres, but the results have been inconsistent. Roberts et al. (Roberts et al., 2014) reported for the first time that leukocyte telomere length (LTL) and the development of AF were not associated when comparing patients with AF to those without AF in the Cardiovascular Health Study (CHS) and they did not obtain evidence based on relative telomere shortening in atrial cells from patients with AF. Consistent results were obtained by (Siland et al., 2017). In contrast, (Carlquist et al., 2016) and (Liu et al., 2021) suggested that telomere shortening is associated with AF and indicated that mitochondrial dysfunction also plays a role in this process. They suggested that the discrepancy in results may be due to differences in the age groups of the selected populations and the study design.

Telomere shortening leads to mitochondrial dysfunction, and the “telomere‐mitochondria‐senescence” axis plays a significant role in this process (Sahin et al., 2011). Telomere loss is linked to mitochondrial dysfunction through three pathways: the “telomere–p53–PGC‐1α/β” axis, the “NAD^+^–SIRT1–PGC‐1α” axis, and the “ATM–AKT–mTOR–PGC‐1β” axis (Zhu et al., 2018). PGC‐1α and PGC‐1β are vital molecules for mitochondrial function because they regulate mitochondrial biogenesis and energy metabolism and are closely related to oxidative stress and inflammation (Sahin et al., 2011). PGC‐1α directly reduces intracellular Ca^2+^ levels and, through the inhibition of oxidative stress, indirectly reduces intracellular Ca^2+^ levels (Eshima et al., 2017). Similarly, in animal experiments, PGC‐1β deficiency can lead to mitochondrial dysfunction, cardiac arrhythmias, and the onset of aging (Ahmad et al., 2018). Telomere shortening activates p53, which inhibits PGC‐1α/β. Shortened telomeres lead to excessive activation of PARP1, resulting in NAD^+^ depletion, which in turn leads to restricted activity of the NAD^+^‐dependent deacetylase sirtuin 1 (SIRT1). Moreover, SIRT1 increases mitochondrial function and biogenesis through the transcription factor PGC‐1α; loss of SIRT1 activity may lead to mitochondrial dysfunction (Zhu et al., 2018). The above evidence suggests that telomere attrition is associated with reduced mitochondrial function. However, a new study indicated that telomere shortening may also impact the aging process by increasing mitochondrial biogenesis. In this study, the researchersproposed that ATM activation by DNA damage activates the mechanistic target of AKT and rapamycin complex 1 (mTORC1), leading to PGC‐1β‐dependent mitochondrial biogenesis and ROS production (Correia‐Melo et al., 2016). Thus, too much or too little mitochondrial synthesis may lead to mitochondrial dysfunction. Mitochondria function optimally only when mitochondrial biogenesis is stabilized. Based on the above studies, telomere shortening induces mitochondrial dysfunction and subsequent reactions through different pathways, including oxidative stress and Ca^2+^ overload, ultimately promoting the occurrence of AF (Peoples et al., 2019). The study by Liu et al. (Liu et al., 2021) further confirmed this view. Using multifactorial regression analysis, they found that LTL (OR 0.365; 95% CI 0.235–0.568; *p* < 0:001) was negatively correlated with the serum PGC‐1α level (OR 0.993; 95% CI 0.988–0.997; *p* = 0:002) and the occurrence of AF; moreover, ROC curve analysis indicated potential diagnostic value for LTL and serum PGC‐1α, with AUCs of 0.734 and 0.633, respectively. They hypothesized that telomeres induce AF by regulating PGC‐1α and subsequent mitochondrial function, oxidative stress, inflammation, and calcium homeostasis, causing atrial electrical remodeling and structural remodeling and ultimately inducing AF.

Certainly, some scholars have proposed that telomere shortening promotes aging by increasing susceptibility to oxidative stress and decreasing genomic stability, followed by fibrosis (Batista et al., 2022; Blasco, 2005). Atrial fibrosis is critical for initiating and maintaining AF because it is a substrate that promotes regional conduction velocity heterogeneity. Heterogeneity in the conduction velocity across regions plays a vital role in the development and maintenance of AF (Burstein & Nattel, 2008). A highly hypothesized relationships exist between a shorter telomere length and autonomic nervous system dysregulation, high sympathetic activation, and a high heart rate (Kim et al., 2019; Révész et al., 2014). Sympathetic and parasympathetic activation is essential for the development of AF. However, unbalanced activation of the sympathetic nervous system characterizes autonomic remodeling in persistent AF. In addition, adrenergic activation‐induced metabolic disturbances provide the setting for acute episodes of AF and facilitate the transition from episodes to sustained stable maintenance of AF (Chakraborty et al., 2023; Chen et al., 2014).

Telomere shortening may contribute to the onset and progression of AF through mitochondrial dysfunction, profibrosis, and the promotion of autonomic remodeling, which is another mechanism for aging‐associated AF.

### 3.3 Disabled macroautophagy

Autophagy is a lysosome‐mediated degradation process that is crucial for protein stabilization by removing potentially toxic cytoplasmic protein aggregates and damaged organelles from cells (Woodall & Gustafsson, 2018). Autophagy is classified into three distinct types: macroautophagy, microautophagy, and chaperone‐mediated autophagy, depending on the mechanism by which cargo is transported to the lysosome (Riquelme et al., 2016). In the discussion below, references to autophagy will mainly refer to macroautophagy (hereafter referred to as “autophagy”) because it plays a crucial role in maintaining myocardial homeostasis and is the best studied category. In autophagy, double‐membrane structures called “autophagosomes” travel along microtubules and engulf damaged organelles and abnormal proteins; they then fuse with lysosomes to form autolysosomes. In the autolysosome, lysosomal hydrolases degrade the cargo into recyclable ATP, amino acids, and fatty acids (Monastyrska et al., 2009). Excessive autophagy induces cardiac remodeling (Li et al., 2015; Maejima et al., 2013; Mialet‐Perez & Vindis, 2017).

The aging‐related decrease in autophagy is one of the most important mechanisms for the decline in organelle turnover, indicating that autophagy is a new hallmark of aging (López‐Otín, Blasco, et al., 2023). Autophagy is crucial for ensuring cardiovascular homeostasis (Sciarretta et al., 2018). During aging, autophagy decreases, and toxic products are not adequately removed or recycled, leading to harmful cardiovascular effects (Shirakabe et al., 2016). In turn, these harmful products contribute to increased oxidative stress, leading to protein and DNA damage, organelle dysfunction, and cell death. Therapeutic strategies that increase autophagy in different models of aging can extend the lifespan and improve cardiovascular function (Sciarretta et al., 2020).

A large body of preclinical evidence suggests that autophagy is a double‐edged sword in cardiovascular disease, playing a beneficial or detrimental role, depending on the circumstances (Bhuiyan et al., 2013). However, the mechanism of autophagy in AF is not well defined, especially in aging‐associated AF. Yuan et al. (Yuan et al., 2018) reported that autophagy flux was significantly activated in a rabbit model exhibiting rapid atrial pacing and in patients with persistent AF, and the activated autophagy flux resulted in a shortened effective atrial ERP and increased susceptibility to AF. They observed that the expression of autophagy‐related gene 7 (ATG7) was upregulated in patients with AF. ATG7 knockdown restored the shortened atrial ERP and attenuated the susceptibility to AF in the animal model. In contrast, ATG7 overexpression significantly increased the incidence and persistence of AF. Autophagy induces atrial electrical remodeling through the ubiquitin‐dependent reduction of selective calcium channels. An increase in AMP‐induced protein kinase (AMPK)‐dependent autophagy was also observed in an early canine model and in atrial cardiomyocytes from patients with AF (Yuan et al., 2014), suggesting that AMPK activation and downstream autophagy may be a novel mechanism involved in AF. Similarly, Wiersma et al. (Wiersma et al., 2017) reported that autophagy was activated in HL‐1 atrial cardiomyocytes after rapid pacing (as evidenced by decreased p62 expression and increased LC3B‐II levels), leading to a decreased Ca^2+^ amplitude. Although SR stress is an upstream activator of autophagy, in vivo treatment with phenyl butyrate sodium salt (an SR stress inhibitor) protects atrial myocytes from electrical remodeling (APD shortening, reduction in L‐type Ca^2+^ currents), cytosolic Ca^2+^ handling/cardiac contractile dysfunction and SR stress and autophagy and attenuates the course of AF. Additionally, activation of CaMKII, a protein involved in the regulation of Ca^2+^ homeostasis, and Ca^2+^ release from the SR can activate the autophagy pathway (Høyer‐Hansen et al., 2007).

Atrial fibrosis is the result of excessive deposition of extracellular matrix (ECM), and atrial fibrosis leads to atrial conduction abnormalities and atrial structural remodeling (mentioned above), both of which predispose individuals to AF (Staerk et al., 2017). Several studies have shown that autophagy is crucial in the regulation of the ECM and that the mechanisms involved are extremely complex. On one hand, autophagy can directly degrade profibrotic factors and thus inhibit fibrosis (Fu et al., 2015); on the other hand, it can modulate the fibrotic process by adjusting the secretion of profibrotic factors (Nüchel et al., 2018). Lin et al. (Lin et al., 2020) found that osteopontin, whose expression is increased in the atrial low voltage zone of AF patients, through the activation of the Akt/GSK‐3β/β‐catenin pathway, inhibits autophagy, thereby reducing the degradation of type I collagen and fibronectin to upregulate atrial fibrosis.

The human body is complex, and aging‐induced autophagy dysfunction may contribute to the onset and progression of AF through electrical remodeling, abnormal Ca^2+^ handling and structural remodeling.

### 3.4 Cellular senescence

Cellular senescence is a state of stable and permanent growth arrest characterized by an inflammatory secretory phenotype (van Deursen, 2014). Cellular senescence can occur due to either acute or chronic injury. Various types of injuries, such as oncogenic signals, genotoxic damage, short telomeres, mitochondrial damage, viral or bacterial infections, oxidative damage, nutrient imbalance, and mechanical stress, can trigger cellular senescence (Gorgoulis et al., 2019). In humans, senescent cells accumulate in multiple tissues at different rates, ranging from 2‐fold to 20‐fold (Tuttle et al., 2019). Cellular senescence is accompanied by changes in cell morphology, chromatin, transcription, metabolism, and secretion (Herranz & Gil, 2018).

Other hallmarks of cellular senescence include the upregulation of p16 and p21 and increased activity of senescence‐associated β‐galactosidase (SA‐β‐Gal) (Caliò et al., 2015). The most favorable evidence for a causal role of cellular senescence in aging is that sustained genetic or pharmacological elimination of senescent cells extends the healthy lifespan of naturally senescent mice (Xu et al., 2018). Focal or tissue‐specific accumulation of senescent cells occurs in many diseases (Zhang et al., 2022). Patients with AF have a significantly increased area of senescent left atrial cells and upregulated SA‐β‐Gal activity and p16 and p21 expression compared with those with sinus rhythm (Adili et al., 2022). SA‐β‐Gal activity and p16 are positively correlated with the degree of atrial fibrosis (Xie et al., 2017).

Cellular senescence leads to the continuous secretion of bioactive molecules that eventually enter the blood through the circulatory system. These molecules are known as senescence‐associated secretory phenotype (SASP) (Coppé et al., 2010). Cellular senescence is closely linked to the activation of the SASP (Calcinotto et al., 2019), which can be initiated through three main pathways: (1) the reduction in the suppression of endogenous retrovirus transcription, causing the release of double‐stranded DNA into the cytoplasm and activating the cGAS/STING and TLR pathways; (2) the overproduction of ROS by mitochondria; and (3) the disturbance of the autophagy‐lysosome system, leading to the accumulation of the lysosomal content and subsequently enhancing the increase in senescence‐associated SA‐β‐Gal activity (López‐Otín, Blasco, et al., 2023). The SASP can be both beneficial and detrimental to organisms. On the one hand, it can be involved in wound healing and immune cell recruitment to eliminate senescent cells (Demaria et al., 2014; Velarde & Demaria, 2016); on the other hand, the accumulation of senescent cells and associated secreted molecules can cause immune senescence and tissue and organ dysfunction (Grubeck‐Loebenstein & Wick, 2002). SASP profiles containing soluble and exosomal SASP factors have recently been established through proteomic analyses of quiescent and replicative fibroblasts and epithelial cells induced by multiple triggers. Among the proteins analyzed, growth differentiation factor 15 (GDF‐15) and matrix MMPs were identified as core SASP proteins (Basisty et al., 2019). In senescent cells induced by overexpression of the RAS oncogene, the top core biomarkers of the SASP, MMPs, and GDF15, are secreted in increased amounts compared with those in controls (Basisty et al., 2019). Atrial fibrosis, a feature of atrial structural remodeling, is associated with the development and maintenance of AF (Lau et al., 2015) (Dzeshka et al., 2015). The SASP is involved in including fibrosis by promoting inflammation and destroying tissue (van Deursen, 2014). Studies have shown that TGF‐β (Verheule et al., 2004), proinflammatory factors (Marcus et al., 2010), and MMPs (Li, 2000) are associated with AF‐related fibrosis. Interestingly, many of these proteins are also SASP factors (Basisty et al., 2019). These results suggest that senescent cells may play a role in AF through inflammatory, fibrotic, and other pathways. Notably, recent studies have proposed that digoxin acts as a protective agent in frail and multimorbid elderly patients by altering the T‐cell pool to ameliorate the proinflammatory SASP (Lee et al., 2023). Based on this information, perhaps we can also provide new avenues for the treatment of aging‐associated AF by inhibiting the cellular senescence pathway.

The previous discussion mentioned the crucial role of SR Ca^2+^ homeostasis in the contraction and relaxation of CM, particularly in subjects with AF. Accumulating evidence indicates that the dysregulation of several SR‐related proteins, including SERCA, RyR2, and PLB, exacerbates the heart dysfunction of AF patients (Herraiz‐Martínez et al., 2015). In a study by (Adili et al., 2022), reduced SERCA2a and RyR2 protein levels and increased levels of PLB were detected in the left auricles of patients with AF (LAAs). In vitro experiments, the same expression pattern was found. However, the SERCA2a and RyR2 levels were restored after the inhibition of cellular senescence. These findings suggest that cellular senescence is also involved in regulating SR‐associated proteins, which may mediate the development of AF through Ca^2+^ instability. However, more experimental data are needed to confirm our hypothesis.

**FIGURE 2:**
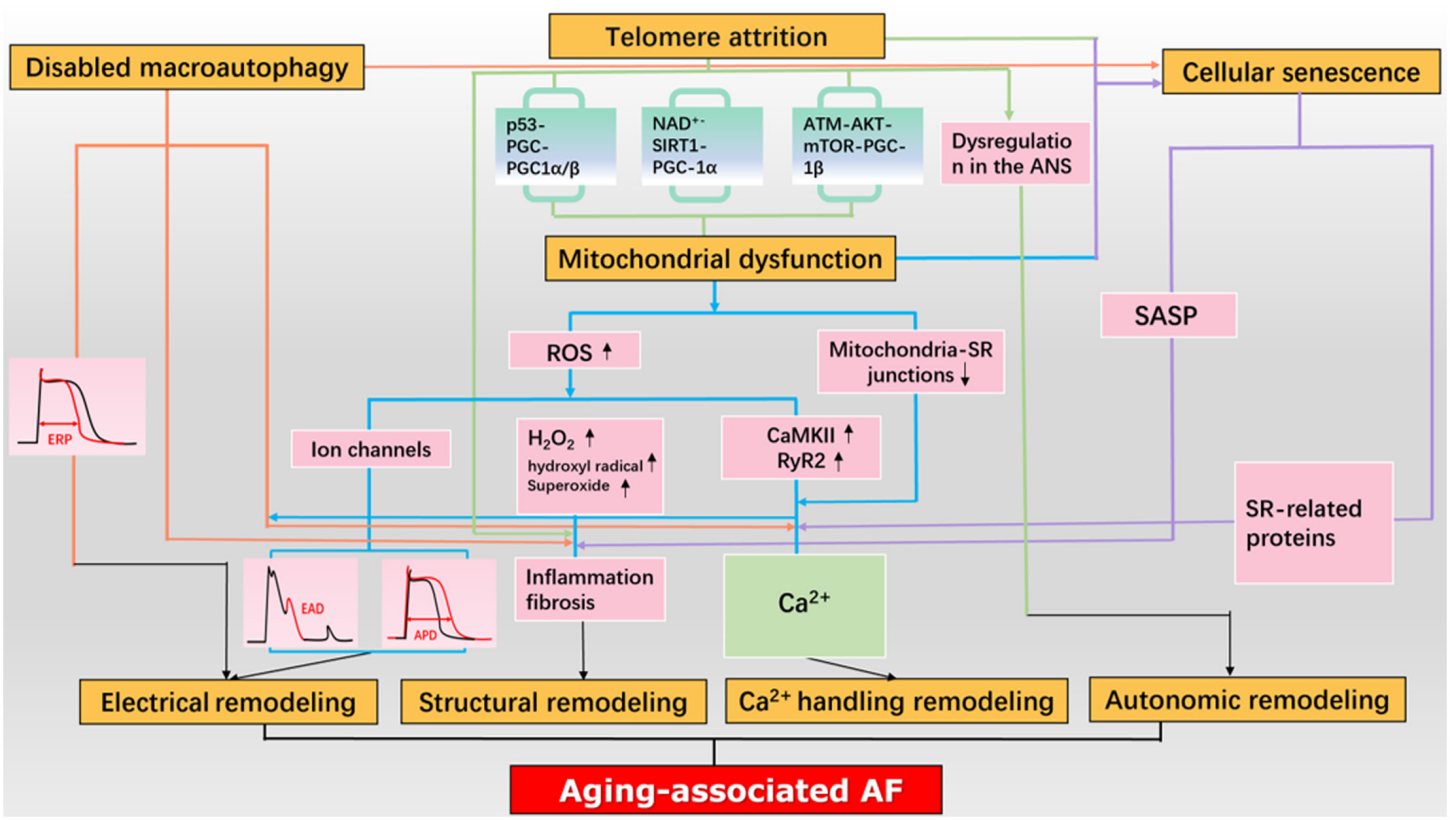
Interrelationships among potential mechanisms of aging‐associated AF. Telomere attrition leads to mitochondrial dysfunction through the activation of three pathways: The “telomere‐p53‐PGC‐1α/β” axis, the “NAD^+^‐SIRT1‐PGC‐1α” axis, and the “ATM‐AKT–mTOR‐PGC‐1β” axis. Shortened telomeres are believed to promote aging by increasing susceptibility to oxidative stress and subsequently leading to fibrosis following a decline in genomic stability. Short telomere length is associated with autonomic nervous system imbalance, with the activation of the sympathetic and parasympathetic nervous systems being crucial for the occurrence of AF. Mitochondrial dysfunction leads to an increase in reactive ROS, which promotes the formation of EADs and prolongs the APD by affecting ion channels. Moreover, an increase in H_2_O_2_, hydroxyl radicals, superoxide, and other ROS can lead to cell inflammation and fibrosis. Elevated ROS levels can enhance CaMKII function, increase RyR2 opening, and induce abnormal Ca^2+^ release, while damage to the connection between mitochondria and the sarcoplasmic reticulum affects Ca^2+^ handling. Increased macroautophagy can result in a shortened atrial effective refractory period. Dysregulated macroautophagy can also lead to disturbances in Ca^2+^ handling and interact with proteins involved in Ca^2+^ homeostasis regulation and sarcoplasmic reticulum Ca^2+^ release. Furthermore, dysregulated macroautophagy can participate in the progression of AF by modulating the fibrotic mechanism. Telomere attrition and mitochondrial damage can both cause cellular senescence, and disruption of the autophagy‐lysosome system resulting in increased lysosomal content can also promote cellular senescence. Cellular senescence affects AF by secreting various inflammatory factors, TGF‐β, and MMPs. It could potentially impact proteins involved in Ca^2+^ homeostasis regulation, ultimately leading to the onset of AF. SIRT1, NAD^+^‐dependent deacetyase sirtuin 1; AF, atrial fibrillation; ANS, autonomic nervous system; ROS, reactive oxygen species; EADs, early afterdepolarizations; APD, action potential duration; H_2_O_2_, hydrogen oxide; CaMKII, calmodulin‐dependent protein kinase II; RyR2, ryanodine receptor isoform 2; ERP, effective refractory period; SR, sarcoplasmic reticulum; TGF‐β, transforming growth factor‐β; MMPs, matrix metalloproteinases; SASP, senescence‐associated secretory phenotype.

Through the above discussion, we found that the potential mechanisms of aging‐associated AF, including classical electrical remodeling, structural remodeling, Ca^2+^ handling remodeling, autonomic remodeling, and recent findings of the involvement of mitochondrial dysfunction, telomere depletion, cellular senescence, and macroautophagy dysfunction are intricately related to each other and finally favor the development and maintenance of AF through interaction (Figure 2).

### 3.5 Gut dysbiosis

**FIGURE 3:**
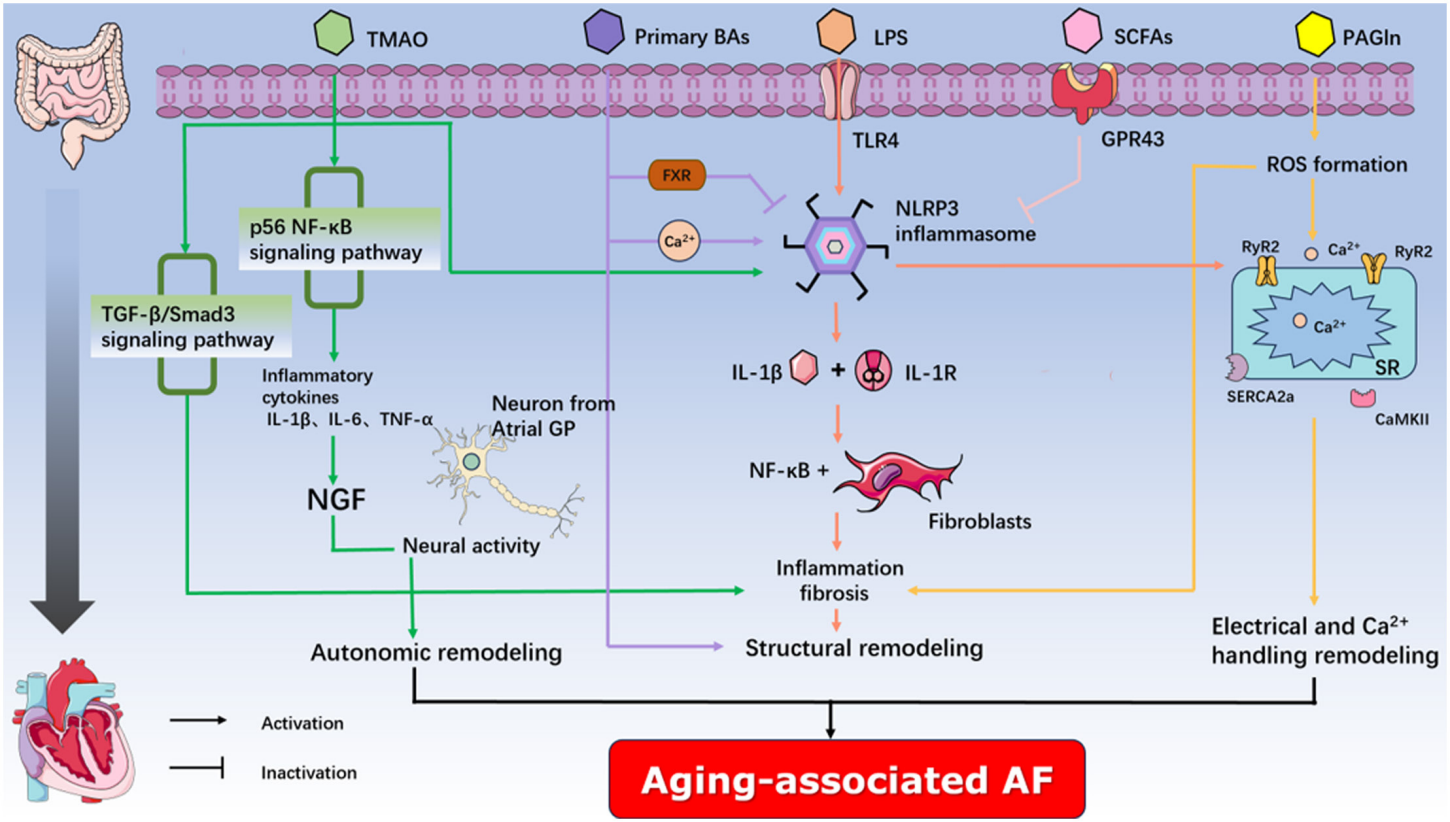
Potential mechanisms of gut dysbiosis and aging‐associated AF. The activation of the NLRP3 inflammasome is initiated by the recognition of LPS by TLR4, which subsequently triggers intracellular signaling cascades. Activation of the NLRP3 inflammasome enhances the release of IL‐1β, leading to the triggering of NF‐κB transcription and myofibroblast differentiation by its binding to IL‐1R, thereby promoting atrial fibrosis. Moreover, the active NLRP3 inflammasome facilitates ectopic electrical activity and reentry in the atria by augmenting the SR Ca^2+^ release mediated by RyR2 and the outward I_Kr_ current in cardiomyocytes. Additionally, TMAO increases proinflammatory cytokines, including IL‐1β, IL‐6, and TNF‐α, by activating the p65 NF‐κB pathway in ARGP, leading to the upregulation of NGF, thereby activating the autonomic nervous system of the heart, ultimately promoting susceptibility to AF. Furthermore, TMAO promotes cardiac fibrosis through the TGF‐β/Smad3 signaling pathway and exacerbates inflammation and cardiac fibrosis by activating the NLRP3 inflammasome. Primary BAs inhibit the activation of NLRP3 inflammasome through the FXR signaling pathway and induce intracellular Ca^2+^ influx to activate NLRP3 inflammasome. CDCA has been demonstrated to induce cardiomyocyte apoptosis and promote structural remodeling, thereby contributing to the progression of AF. SCFAs attenuate the activity of NLRP3 inflammasome through GPR43‐mediated inactivation of NLRP3, thereby mitigating the progression of AF. PAGln increases cellular apoptosis, ROS production, CaMKII, and RyR2 activation, and decreases cell viability. The accumulation of ROS promotes CaMKII and RyR2 activation, Ca^2+^ release, and calcium overload. ROS also promotes atrial fibrosis and inflammation, which are essential drivers of atrial structural remodeling and provide the substrate for AF. Additionally, calcium overload is also considered an important mechanism underlying the occurrence of AF. AF, atrial fibrillation; LPS, lipopolysaccharide; TLR4, toll‐like receptor 4; NLRP3, NACHT, LRR, and PYD domains‐containing protein 3; IL‐1R, interleukin‐1 receptor; IL‐1β, interleukin‐1β; IL‐6, interleukin‐6; NF‐κB, nuclear factor kappa B; TGF‐β, transforming growth factor β; SR, sarcoplasmic reticulum; TAMO, trimethylamine N‐oxide; ARGP, anterior right ganglionated plexi; GP, ganglionated plexi; NGF, nerve growth factor; SCFAs, short‐chain fatty acids; CDCA, chenodeoxycholic acid; BAs, bile acids; PAGln, phenyl acetyl glutamine; RyR2, ryanodine receptor isoform 2; ROS, reactive oxygen species; GPR43, G‐protein‐coupled receptor 43; SERCA2a, SR Ca^2+^‐ATPase type 2a; CaMKII, calmodulin‐dependent protein kinase II:GPR43, G‐protein‐coupled receptor 43.

The microorganisms that inhabit the human gut are referred to as the “gut microbiota (GM)”, and as we age, the composition, diversity, and function of the GM differ between young and old individuals, which has been referred to as gut dysbiosis in elderly individuals (Ling et al., 2020). A growing body of evidence suggests that gut microbes play a crucial role in regulating healthy aging and ultimately influence host longevity (Bárcena et al., 2019; Biagi et al., 2016). In animal experiments, the healthy lifespan and longevity of prematurely aged mice were extended through fecal microbiota transplantation (FMT) (Bárcena et al., 2019). More recently, an experimental study revealed that transferring microbiota from older to younger mice accelerates aging‐associated signaling, retinal inflammation, and central nervous system inflammation. Fortunately, these harmful effects can be reversed by transferring the microbiota of young mice (Parker et al., 2022). Aging‐associated changes in the composition, diversity, and functionality of gut microbes, with consequent chronic inflammation and immunosenescence, are the pathophysiological basis of many aging‐related diseases. The GM and its metabolic products, including lipopolysaccharides (LPS), trimethylamine N‐oxide (TMAO), bile acids (BAs), short‐chain fatty acids (SCFAs), and phenylacetylglutamine (PAGln), play critical roles in age‐related cardiovascular diseases (Ling et al., 2020). Interestingly, recent evidence also suggested a potential link between gut dysbiosis and AF (Figure 3).

LPS, a gram‐negative bacteria cell wall component, is a potent trigger of systemic inflammation in the host. LPS levels increase with the aging process (Zhang, Zhang, et al., 2021). LPS disrupts the integrity of the intestinal barrier (Guo et al., 2015), leading to intestinal “leakiness” and chronic inflammation. A recent analysis of macrogenomic data has shown that microorganisms involved in LPS synthesis are clustered in the gut of patients with AF and that the gene encoding LPS synthase is upregulated (Zuo, Zhang, et al., 2022). Elevated serum LPS levels have been reported to predict adverse events in patients with AF (Pastori et al., 2017). (Zhang, Zhang, et al., 2021) transplanted the microflora from older rats into the intestines of younger rats in a fecal microbial transplantation (FMT) rat model and found that circulating levels of LPS and glucose were increased in young hosts, resulting in increased levels of ‘NACHT, LRR and PYD domains‐containing protein 3’ (NLRP3) inflammasome expression, which ultimately led to enhanced atrial fibrosis and AF susceptibility in young hosts. Interestingly, these changes could be reversed by recolonizing the immature microbiota. Indeed, LPS can be translocated from the gut into the circulation via paracellular or transcellular mechanisms and is rapidly recognized by toll‐like receptor 4 (TLR4) (Lu et al., 2008). TLR4 subsequently activates intracellular signaling cascades, leading to the activation of the NLRP3 inflammasome (Yao et al., 2018). Additionally, recent studies have shown that the NLRP3 inflammasome is activated in the atria of patients with AF and is involved in the development of AF (Yao et al., 2018). This evidence was established in a mouse model with a specific knock‐in of NLRP3 in CM, called CM‐KI, which constitutively expresses NLRP3. Spontaneous premature atrial beats and inducible AF occurred in the CM‐KI mouse model. Abnormal Ca^2+^ release from the SR, a shortened atrial ERP, and cardiac hypertrophy were observed (Yao et al., 2018). The activated NLRP3 inflammasome promotes ectopic electrical activity and reentry by enhancing RyR2‐mediated SR Ca^2+^ release and outward currents in cardiomyocytes (Li & Brundel, 2020). Activation of the NLRP3 inflammasome also leads to the release of the cytokine interleukin‐1β (IL‐1β), which, in combination with the IL‐1 receptor, can trigger the nuclear factor kappa B (NF‐κB) transcription and myofibroblast differentiation, leading to atrial fibrosis (Li & Brundel, 2020). The specific NLRP3 inflammasome inhibitor MCC950 can reduce AF susceptibility and atrial fibrosis in a rat model (Zhang, Zhang, et al., 2021). Thus, LPS increases the susceptibility to AF through the remodeling of atrial electrical, structural, and Ca^2+^ handling by the NLRP3 inflammasome, etc. In addition to an increase in LPS levels with age (Zhang, Zhang, et al., 2021), recent studies have shown that LPS can directly induce cellular senescence and increase the inflammatory profile of senescent endothelial cells, therefore, this study proposes that the inhibition of LPS may offer a new approach for treating senescence‐related diseases (Suzuki et al., 2022).

TMAO is a critical product of the GM. TMAO levels are reportedly elevated in elderly individuals, and high TMAO levels are associated with endothelial senescence and vascular aging (El Hage et al., 2023). In a 2018 study, an elevated serum TMAO level was positively associated with long‐range AF. After adjusting for potential confounding covariates, this association was unrelated to traditional AF risk factors (Svingen et al., 2018). Thus, circulating TMAO levels may be an independent risk factor for the development of AF. This finding was further validated in a macroeconomic data mining analysis (Zuo, Fang, et al., 2020). The specific mechanism by which TMAO induces AF is not particularly clear. However, it can be summarized as follows: (1). Cardiac autonomic nervous system activation. Several scholars have shown increased expression of nerve growth factor (NGF) in a canine model of AF induced by rapid atrial pacing in the ganglionated plexi (GP) that leads to electrical remodeling of the atria, thereby creating a substrate for AF (Zhou et al., 2020). Yu et al. (Yu et al., 2018)injected TMAO locally into four main atrial ganglion plexuses (GPs), leading to marked activation of the right anterior GP (ARGP) function and nerve activity, as well as increased atrial electrical instability. These changes are mainly attributed to TMAO activating the proinflammatory p65 NF‐κB signaling pathway, leading to increased expression of inflammatory cytokines (including IL‐1β, IL‐6, and TNF‐α), which in turn triggers the instability of the atrial rhythm. (2). Cardiac remodeling and cardiac fibrosis. TMAO exacerbates cardiac fibrosis in mice at least partially by activating the NLRP3 inflammasome and increasing oxidative stress in induced cultured cardiac fibroblasts. The data from this study suggest that TMAO exacerbates cardiac fibrosis in mice, at least in part through the activation of the NLRP3 inflammasome. This study also revealed that TMAO dose‐dependently increased cardiac fibrosis by promoting fibroblast proliferation, migration, and collagen secretion through TGF‐β/Smad3 signaling (Li et al., 2019). In addition, another study showed that 3,3‐dimethyl‐1‐butanol (DMB) reduced plasma TMAO levels and attenuated pressure overload‐induced cardiac hypertrophy, cardiac fibrosis, and ventricular arrhythmias by inhibiting the p65 NF‐κB and TGF‐β1/Smad3 signaling pathways, which may further substantiate the role of TMAO in cardiac remodeling (Wang et al., 2020). (3). Inflammation and oxidative stress. Several studies have shown that increased serum TMAO concentrations significantly contribute to the inflammatory response through various pathways (Li et al., 2019). To the best of our knowledge, multiple inflammation‐activated signaling pathways may play an essential roles in the development of AF. However, the specific pathways that are the leading causes of AF have not been fully elucidated (Ajoolabady et al., 2022). TMAO promotes AF through cardiac autonomic remodeling, structural remodeling, inflammation, and oxidative stress.

Age‐related changes in BA composition in the bile, liver, and serum have been reported in mammals. Although the nature of these changes varies according to many factors, aging is primarily associated with decreased BA levels (Perino et al., 2020). Further supporting this finding is the fact that longer‐lived pygmy mice (Ghrhr^lit/lit^), which are characterized by defective growth hormone/IGF‐1 signaling, have an increased biliary pool size (Amador‐Noguez et al., 2007). Furthermore, the use of CA in wild‐type mice mimics the changes in drug‐metabolizing enzymes observed in Ghrhr^lit/lit^ mice, suggesting that BA‐induced xenobiotic responses may contribute to longevity (Amador‐Noguez et al., 2004). BAs have also been shown to influence arrhythmogenic mechanisms. Primary BAs (e.g., chenodeoxycholic acid CDCA) form bile salts by binding to amino acids (taurine or glycine) and are further secreted into the small intestine. Taurine‐bound BAs can induce changes in membrane potential via cardiac sodium‐calcium exchange (Rainer et al., 2013) and activate I_KACh_ in cardiomyocytes, which may promote AF (Sheikh Abdul Kadir et al., 2010). In the gut, primary BAs are not bound by bile salt hydrolases produced by the GM. In the presence of colonic bacteria, they can be further transformed by dehydrogenation, dihydroxylation, or isomerization to form secondary BAs (e.g., ursodeoxycholic acid). The dysregulated intestinal flora regulates the BA ratio such that the concentration of secondary BAs (e.g., ursodeoxycholic acid) decreases while that of primary BAs (e.g., chenodeoxycholic acid) increases. Although chenodeoxycholic acid has been shown to cause cardiomyocyte apoptosis and promote structural remodeling, which contributes to the progression of AF (Wang et al., 2019), this process can be exacerbated by the inflammatory process through NLRP3 inflammasome activation (Gong et al., 2016). The Secondary BA ursodeoxycholic acid plays a role in preventing arrhythmia by stabilizing the cell membrane potential (Zhu et al., 2020). The pro‐AF effect of primary BAs and the protective effect of secondary BAs were also confirmed in a study by Rainer et al. They found that taurocholic acid (TCA) concentration‐dependently induced atrial arrhythmias in mice. In addition, patients with AF show significantly reduced serum levels of ursodeoxycholic acid conjugates and high levels of nonursodeoxycholic acid, both of which contribute to a lower threshold for arrhythmia development, thereby promoting the occurrence of arrhythmias (Rainer et al., 2013). BAs were shown to bidirectionally regulate the activity of the NLRP3 inflammasome. BAs have been shown to inhibit NLRP3 activation through FXR signaling, whereas BAs‐induced Ca^2+^influx activates the NLRP3 inflammasome (Perino et al., 2020). Researchers have also proposed that the bidirectional regulatory effect of BAs on NLRP3 inflammasome vesicles depends on whether the organism is under inflammatory conditions (Liao et al., 2022). Therefore, BAs, primarily primary BAs, affect electrical activity by regulating ion channels; BAs also alter the myocardial structure by regulating inflammasomes and their effects on apoptosis, ultimately increasing the onset and maintenance of AF. Of course, the mechanisms involved are intricate and deserve more in‐depth study.

SCFAs are the primary metabolites produced by the colonic flora from the breakdown of dietary fiber. SCFAs reduce systemic inflammation, improve arterial compliance, and regulate blood pressure; they also enhance intestinal barrier function, counteract endotoxemia, and protect against atherosclerosis, all of which suggest that SCFAs play a role in improving cardiac function and maintaining cardiovascular homeostasis (Hu et al., 2022). SCFAs have also been shown to prolong thelifespan, and Smith et al. observed changes in the gut microbiome and fermentation products (e.g., SCFAs) in acarbose‐treated mice, along with a prolonged lifespan (Smith et al., 2019). Therefore, researchers have theorized that SCFAs are negatively correlated with the development of AF. Several studies have evaluated the relationship between SCFAs and AF. A metagenomic data mining analysis of a northern Chinese population revealed that microbial genes involved in SCFA synthesis were significantly reduced in the gut of AF patients, and dysbiosis of the gut microflora in AF patients was associated with the disruption of genes related to SCFA synthesis (Zhang, Zuo, et al., 2021). A study by Zuo et al. (2022) revealed that the fecal SCFA levels were significantly reduced in AF patients. In a mouse model lacking dietary fiber, a decrease in SCFA‐producing probiotics, a decrease in plasma SCFA levels, a prolongation of the P‐wave duration, an increase in the left atrial internal diameter in mice, activation of the NLRP3 inflammasome, and an increase in susceptibility to AF were observed. SCFA supplementation prevented the phosphorylation of CaMKII and RyR2, which were upregulated by dietary fiber deficiency, and prevented fibrosis, collagen expression, and NLRP3 inflammasome activation in atrial tissue. Finally, it was confirmed by in vitro experiments that SCFAs attenuate the activity of NLRP3 inflammasome through G‐protein‐coupled receptor 43 (GPR43)‐mediated inactivation of NLRP3, thereby alleviating the progression of AF. SCFAs are postulated to delay AF through multiple pathways, such as inhibiting inflammation, regulating Ca^2+^ homeostasis, and improving atrial myocardial remodeling. However, extensive in vivo and in vitro research to verify our speculation.

PAGln is also a metabolite of GM. According to metagenomic data, (Fang et al., 2022) reported that the relative abundance of the porA enzyme, an essential bacterial enzyme for PAGln synthesis, tended to increase in feces from patients with AF and plasma levels of PAGln were significantly increased in patients with AF. In addition, the PAGln level was positively correlated with left atrial internal diameter. Subsequently, in vitro experiments revealed that PAGln increased apoptosis, ROS production, and CaMKII and RyR2 activation and decreased cell viability. The accumulation of ROS promotes the activation of CaMKII and RyR2, Ca^2+^ release, and calcium overload (Ren et al., 2018). All of these processes are associated with AF, as described in detail above. Oxidative stress and cell apoptosis together promote atrial fibrosis and inflammation, serving as important driving forces for atrial structural remodeling and providing the material basis for the occurrence of AF (Aimé‐Sempé et al., 1999; Karam et al., 2017). Therefore, PAGln may be involved in the pathogenesis of AF by promoting Ca^2+^ release, oxidative stress, and apoptosis in atrial myocytes. Whether aging‐associated intestinal microecological dysbiosis alters PAGln levels, thereby increasing the occurrence of AF, has not been addressed in current studies, and more prospective studies are needed to fill this gap.

## 4 CONCLUSIONS

A growing body of evidence suggests that inhibiting aging‐related pathways provides new avenues for the treatment of age‐related diseases (Abraham & Li, 2022), and aging is an important factor in the development of AF. The mechanisms of aging‐associated AF include electrical remodeling, structural remodeling, and Ca^2+^ handling remodeling, which are widely known. Moreover, recent studies have suggested other triggers associated with aging including mitochondrial dysfunction, telomere attrition, cellular senescence, macroautophagy dysfunction, and gut dysbiosis. These novel mechanisms play a role in the pathophysiology of age‐related diseases (López‐Otín, Blasco, et al., 2023), so thus further elucidation of the role of these processes in the development and maintenance of AF is necessary to understand the mechanisms of AF in all areas of aging. For example, telomere depletion can cause mitochondrial dysfunction through three pathways (Zhu et al., 2018), and mitochondrial dysfunction can increase susceptibility to AF through increased oxidative stress (Youn et al., 2013). Telomere depletion, mitochondrial dysfunction, and increased macroautophagy are all factors involved in cellular senescence (López‐Otín, Blasco, et al., 2023) (Gorgoulis et al., 2019), and cellular senescence further impacts AF through inflammatory factors (Marcus et al., 2010) and dysfunction of Ca^2+^‐related proteins (Adili et al., 2022). Dysbiosis of the intestinal flora and consequent metabolite abnormalities can increase susceptibility to AF through autonomic remodeling, structural remodeling, electrical remodeling, and Ca^2+^ handling remodeling (Gawałko et al., 2021). Aging‐associated AF is a disease whose mechanisms have not been clearly defined, and thus investigating the mechanisms of AF through all levels of aging‐associated features may provide additional approaches for the treatment of elderly patients.

## AUTHOR CONTRIBUTIONS

MFW, CH, FJ, and CHZ were involved in conceptualizing and designing this work; when disagreements arose, the four authors (MFW, CH, FJ, and CHZ) discussed and resolved the disagreements.CX and JJL were involved in the drafting of the manuscript, reviewing and providing critical feedback, and critically revising important intellectual content. All authors read and approved the final version and are responsible for the published content. All authors participated fully in the work and agreed to take responsibility for all aspects of the work.

## FUNDING INFORMATION

The study was supported by grants from Changzhou Key Medical Discipline (CZXK202202), Changzhou Sci&Tech Program (CJ20235088), and Changzhou Sci&Tech Program (CJ20235085).

## CONFLICT OF INTEREST STATEMENT

We declare no conflicts of interest.

## Data Availability

I confirm that my article contains a Data Availability Statement even if no new data was generated (list of sample statements) unless my article type does not require one.
